# The role of immunoglobulin E in non-atopic disorders

**DOI:** 10.3389/fimmu.2025.1728940

**Published:** 2026-01-06

**Authors:** Kujtim Thaçi, Aron Gyorgypal, Robert M. Anthony, Michelle E. Conroy

**Affiliations:** 1 Center for Immunology and Inflammatory Diseases, Massachusetts General Hospital, Harvard Medical School, Boston, MA, United States; 2 Department of Medical Biochemistry, University for Business and Technology (UBT)-Higher Education Institution, Prishtina, Kosovo

**Keywords:** autoimmune diseases, cancer, IgE glycosylation, immunoglobulin E, parasitic infections

## Abstract

Immunoglobulin E (IgE) and its corresponding Fc epsilon receptors (FcϵRs) are essential components of the immune system. The constant, crystallizable fragment (Fc) region of IgE binds with high affinity to its specific receptor, FcϵRI, anchoring IgE molecules to the surface of effector cells such as mast cells and basophils. Once bound, IgE uses its antigen-binding fragment (Fab) to recognize specific antigens. Antigen-induced crosslinking of cell-bound IgE triggers activation of these effector cells. Over fifty years ago, intensive research identified IgE as a key mediator of allergic reactions. Subsequent studies have demonstrated that the production of antigen-specific IgE and its interactions with innate immune cells are critical not only for allergic responses but also for certain non-atopic immune processes. N-glycosylation, a crucial post-translational modification, has been shown to strongly influence the stability and function of IgG antibodies. Similarly, glycosylation is vital for maintaining the structure and biological activity of IgE. Individual variations in IgE glycosylation patterns regulate its functional properties, contributing to the diversity and complexity of IgE-mediated immune responses. Given the emerging role of IgE in non-atopic diseases, understanding how site-specific glycosylation variations affect IgE function is essential for characterizing disease-specific molecular signatures and identifying new therapeutic targets. Comprehensive glycoproteomic analyses of IgE from diverse pathological conditions may clarify how glycosylation influences disease progression, identify Fc glycans associated with pathology, and elucidate their biological roles.

## 1 Introduction

The year 2016 marked the 50th anniversary of the discovery of immunoglobulin E (IgE) (1, 2). Since then, the relationship between immunoglobulin (Ig) structure and function has been extensively studied. However, the low serum concentration of IgE and the historical lack of sensitive analytical methods have limited a full understanding of its biology. The availability of large quantities of IgE from individuals with myeloma facilitated both its discovery (3, 4) and the development of the first-generation assays for antigen-specific IgE (5). More recent studies employing X-ray crystallography, nuclear magnetic resonance (NMR), and other biophysical techniques have elucidated IgE binding sites and kinetics, providing new insights into its structural and functional characteristics.

IgE antibodies play a central role in mediating immediate hypersensitivity reactions, including urticaria, bronchospasm, systemic anaphylaxis, and chronic inflammatory disorders such as rhinitis, atopic dermatitis, and asthma (6). The critical mechanism underlying these responses involves allergen-induced crosslinking of IgE bound to high-affinity FcϵRI receptors on effector cells such as mast cells (MCs), basophils, and eosinophils. This interaction triggers the release of cytokines and inflammatory mediators, including histamine, heparin, tryptase, and prostaglandins (3).

Beyond allergic disease, IgE contributes to host defense against parasitic infections (7–9) and protection from venom toxins (10) (11–14). Emerging evidence also suggests a potential role for IgE in anti-tumor immunity (15–17), autoimmunity (18–21), and respiratory viruses (22–25), underscoring its broader relevance in immune regulation.

This review aims to summarize current knowledge of IgE’s structural and functional properties and to explore its potential protective roles in host defense and non-atopic pathological conditions.

## 2 The structure of immunoglobulin E and its receptors

IgE is the least abundant immunoglobulin class in serum, with concentrations ranging from 150 to 300 ng/mL (26–28)—approximately a thousand-fold lower than IgG, which averages around 10 mg/mL (29). This extremely low concentration reflects the tight regulation of IgE production and secretion through complex molecular and cellular mechanisms.

Studies of interleukin-4 (IL-4) and interleukin-13 (IL-13) signaling have provided key insights into the regulation of IgE synthesis. When IL-4 and IL-13 engage their receptors on B cells, they activate the Janus kinase 3 (JAK3) and signal transducer and activator of transcription 6 (STAT6) pathways (30), promoting immunoglobulin class-switch recombination (CSR) and inducing IgE production (31–33).

**Figure 1:**
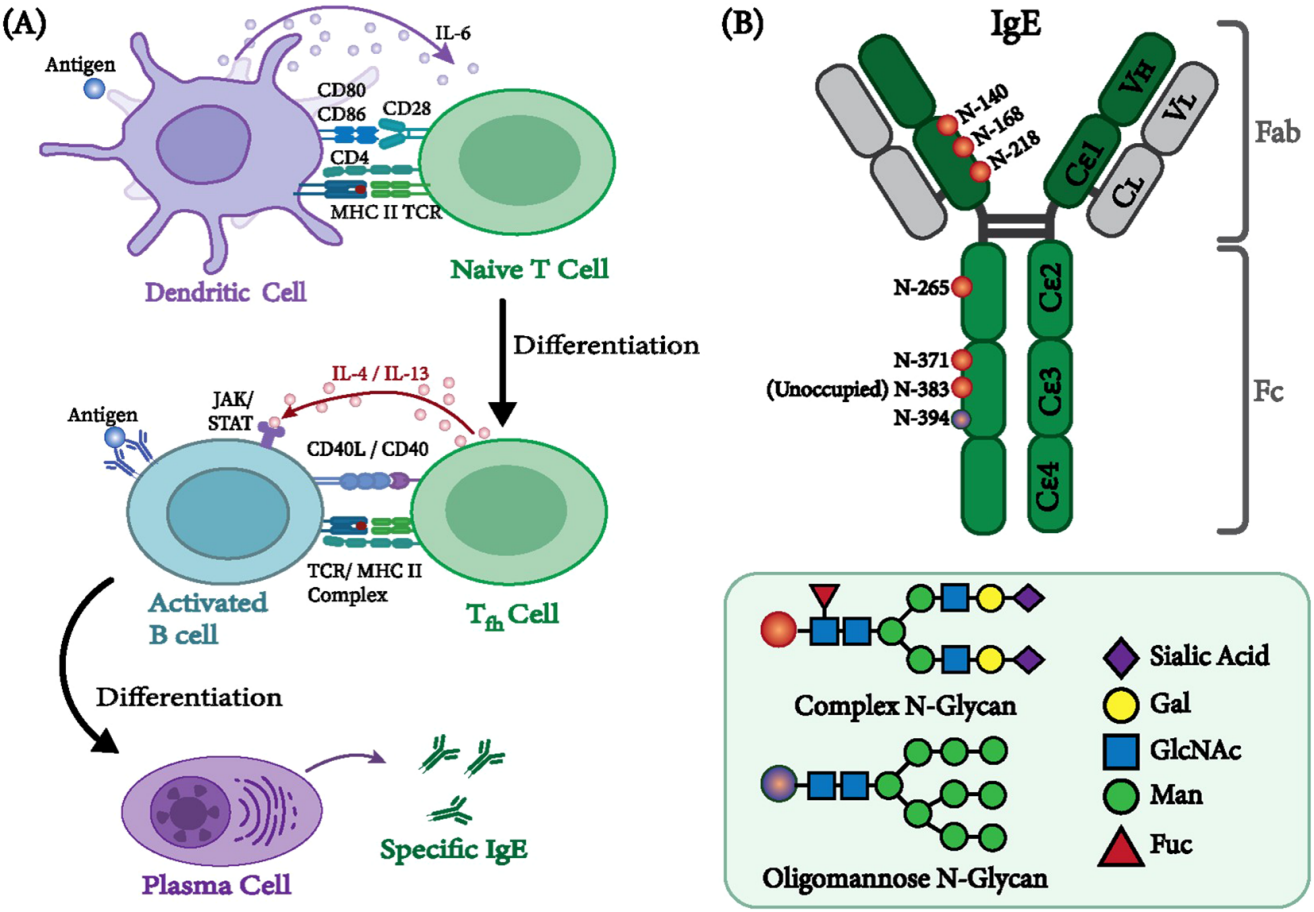
Human immunoglobulin E and its site-specific glycosylation **(A)** Regulation of immunoglobulin class switching to IgE. DCs attach antigens and present them to native T cells via major histocompatibility complex (MHC) class II molecules. Following the activation and proliferation of naive T cells, they transform into Th2 cells. MHC class II molecules facilitate the interaction between B cells and their membrane receptors. Activated Th2 cells trigger the production of IL 4 and IL 13, which elicit IgE synthesis in immature human B cells (31, 32). The mechanism behind this interplay between cells is facilitated by the interaction between cytokines and their receptors, which trigger a signaling cascade involving JAK3 and STAT6 (30). The CD40 receptor on the B-cell interacts with the CD-40 ligand (CD-40L) on the T-cell, another signal required for the switch from isotype Ig to isotype IgE. **(B)** The structural and glycosylation aspects of IgE are schematically illustrated. The IgE-Fc site-specific glycosylation is shown with closed and open circles, representing complex and oligomannose glycans. Complex-type glycans consist of fucose (red), GlcNAc (blue), mannose (green), galactose (yellow), and sialic acid (pink). Oligomannose-type glycans comprise N-acetylglucosamine (GlcNAc) and various mannose residues, typically ranging from 5 to 9.

An additional co-stimulatory signal is required for efficient switching to IgE. This occurs through the interaction between CD40 on B cells and CD40 ligand (CD40L) expressed on activated T cells (**Figure 1a**) (34). Upon activation, T cells upregulate CD40L and secrete IL-4 and IL-13, which together induce transcription of the ϵ heavy-chain gene and initiate IgE class switching in B cells (32, 35–37). Th2 cells drive CSR from IgM^+^ or IgG^+^ B cells primarily within germinal centers (GC) of secondary lymphoid tissues. Respectively, follicular helper T (Tfh) cells promote B cell proliferation, affinity maturation, and differentiation into high-affinity IgE-producing cells within GCs (38–40). Among these, IL-4–and IL-21–producing Tfh subsets regulate immunoglobulin class switching and recombination (41). In contrast, Tfh13 cells, a subset of Tfh cells that produce IL-13, drive the production of high-affinity IgE involved in allergic responses and anaphylaxis (39, 42). Hence, targeting Tfh13 cells could offer a different therapeutic approach to reduce the severity of anaphylaxis.

The differentiation of Tfh cells goes through three distinct developmental stages: an initial phase resembling progenitor cells, a fully developed effector phase, and a post-effector Tfh phase that maintains transcriptional and epigenetic traits but does not produce IL-21 (43). Data indicate that the transcription factor FoxP1 plays a crucial role in regulating the progression of Tfh through all these stages, while follicular regulatory T cells (Tfr) provide an extrinsic regulatory mechanism, which is thought to suppress GC B cells and the antibody response (43, 44). Researchers reported that selectively deleting all stages of Tfh cells influenced antibody dynamics at different points during the germinal center reaction in response to a SARS-CoV-2 vaccine (43).

Conspicuously, Tfr cells, previously thought to mainly suppress antibody responses, these cells have a critical helper function in promoting food antigen-specific IgE production (45, 46). Specifically, the helper function of Tfr cells is mediated by the cytokines IL-10 and IL-4 (44). Notably, loss of IL-10 signaling in B cells resulted in severely reduced peanut-specific IgE, decreased GC B cell survival, and loss of GC dark zone B cells after allergen sensitization (45, 46). Furthermore, in the mouse model, IL-1R2 deficiency led to greater IL-1R1-dependent Tfr cell activation and expansion (47). Data have indicated that Tfr cells suppressed GC B cell growth and IgG production but allowed strong IgE responses, likely by increasing IL-4+ Tfh cells (47). Together, these findings revise the traditional view of Tfr cells as suppressive, showing they are essential for the development of food antigen-specific IgE and thus play a direct role in food allergy pathogenesis. As a result, targeting Tfr cell-derived IL-4 and IL-10 could be a novel approach for food allergy therapies.

Although distinct stages of Tfh differentiation have been described, the exact mechanism by which a subset of activated CD4+ T cells triggers CXCR5 expression during the early immune response is unclear (48). Moreover, the factors regulating the migration of defined CXCR5+ precursor Tfh (pre-Tfh) cells into B cell follicles within the GC, and their maturation into germinal center Tfh (GC-Tfh) cells, remain insufficiently characterized (48). In contrast, other activated CD4+ T cells pursue divergent developmental paths. The data provided further insight into the differentiation of Tfh cells and their essential function in strengthening humoral immunity. Therefore, understanding the differentiation of Tfh cells is essential for advancing vaccine development, treating autoimmune disorders, and improving cancer immunotherapy (41, 49, 50). Targeting Tfh cells therapeutically is promising but complex, underscoring the need for precise strategies that maximize their benefits while minimizing risks.

Moreover, type 2-polarized memory B cells (MBC2s), characterized by high expression of CD23 and IL-4Rα, and low expression of CD32, have been shown to contribute to the production of allergen-specific IgE in the bloodstream during sublingual immunotherapy for patients with allergic rhinitis and food allergy (51). These cells serve as a major reservoir and an essential source of IgE (51). Investigation of MBC2’s function offers important insights into the persistence of IgE memory, which is detrimental in allergic conditions but may provide protection against venom and helminth infections.

Although CSR was long thought to be restricted to these germinal centers, evidence now indicates that local classes switching to IgE can also occur at sites of allergic inflammation (52). Several studies suggest that mucosal tissues may serve as additional sites for somatic hypermutation and IgE class switching (53–57). Indeed, local production of IgE^+^ B cells and IgE^+^ plasma cells have been detected in the nasal mucosa of patients with seasonal and perennial allergic rhinitis (58).

Interestingly, natural IgE production can also occur independently of T cells and germinal centers. In T-cell-deficient and germ-free wild-type mice, IgE synthesis proceeds through mechanisms not dependent on MHC class II (MHC II), though IL-4 may still play a role (59). These naturally produced IgE antibodies can recognize self-antigens and are not necessarily inhibited by regulatory T cells (59).

During the Th2 immune response, activated B cells extend their role beyond producing antibodies. These cells can differentiate into effector B cells (Be2), which secrete IL-4 along with other cytokines (60). These cytokines help in the differentiation of naive CD4+ T cells into Th2 cells (61–63). Subsequently, IL-4 produced by Th2 cells triggers immunoglobulin class switching to IgE on B-cells, which further differentiate into plasma cells (64, 65). This interaction is a reciprocal mechanism of action between these two hematological immune cells that drives specific adaptive immunity responses against various intrinsic and extrinsic antigens.

Although IL-4 and IL-13 are primary cytokines that trigger CSR, reports suggest that IFN-γ, TGF-β, and IL-21 inhibit CSR by directly suppressing germline transcription or antagonizing IL-4 signaling (66). Moreover, TGF-β impairs Tfh2 development via the PI3Kγ/mTOR pathway, thereby protecting against allergic diseases (67). Consequently, balancing these stimulatory and inhibitory signals is crucial for the precise regulation of IgE production and secretion.

Two major forms of IgE are present in circulation: membrane-bound IgE (mIgE), expressed on B cells, and soluble IgE (sIgE), the secreted form found in serum (68, 69). Free sIgE binds to high-affinity Fcϵ receptors (FcϵRs) on effector cells, leading to their sensitization (70). The half-life of circulating sIgE is approximately two days—much shorter than that of IgG, which persists for about 21 days (71). However, IgE exhibits a prolonged tissue half-life, lasting weeks to months, due to its stable binding to FcϵRI on effector cells (72).

Structurally, IgE shares the basic immunoglobulin architecture with other antibody classes, consisting of two identical heavy (H) and two light (L) chains linked by disulfide bonds. Each molecule contains two antigen-binding Fab fragments—each composed of one variable (V) and one constant (C) domain—and a crystallizable Fc region. The Fc portion consists of four constant domains (Cϵ1–Cϵ4) and lacks the hinge region found in IgG, which has only three constant domains (CH1–CH3). While the IgE Fab portion binds to antigens and provides the structural framework for the immense immunological diversity of antibodies, the Fc portion induces potent effector functions (73). IgE elicit effector functions by binding to effector cell receptors, the high-affinity FcϵRI receptor, and the low-affinity FcϵRII/CD23 receptor. The FcϵRI receptor consists of four subunits: α, β, and a homodimeric γ subunit. The FcϵRIα binds to the Fc region of IgE (Fcϵ), whereas the FcϵRIβ and FcRγ subunits are integral to signal transduction (74–76). Cryo-electron microscopy (cryo-EM) structures of both the apo state of FcϵRI and FcϵRI bound to Fcϵ reveal that signal transduction is facilitated by intracellular immunoreceptor tyrosine-based activation motifs (ITAMs) (76). IgE binding stabilizes receptor conformation and elucidates how receptor clustering or crosslinking by multivalent allergens results in Syk activation and subsequent degranulation (77, 78). Transmembrane interactions among the α, β, and γ subunits determine the spacing and orientation of intracellular ITAMs, which influence Lyn and Syk recruitment and the strength of downstream Ca²^+^ and MAPK signaling (76, 78, 79). Moreover, ubiquitin-specific protease 5 (USP5) regulates FcϵRIγ stability in mast cells by determining whether it is degraded or stabilized (80). Thus, the USP5-FcϵRIγ interaction could be a therapeutic target for reducing allergic responses (80).

FcϵRI receptor is structurally identical to other members of the FcγR family, while the FcϵRII/CD23 receptor belongs to the C-type (Ca2+-dependent) lectin-like superfamily (81). The absence of a hinge and the presence of the Cϵ2 domain give IgE a more rigid and asymmetrically folded conformation, resulting in lower flexibility compared with IgG (7, 82, 83). IgE maintains a bent structure both in solution and when bound to its high-affinity receptor FcϵRI (84–86).

The Cϵ2 domains are believed to contribute to the prolonged stability of the IgE–FcϵRI complex (87) by stabilizing receptor interactions through conformational changes (88). In contrast to the lower affinity observed in IgG’s interaction with FcγR (Kd≈10–^6^ to 10–^8^ M), IgE exhibits a unique 1:1 binding ratio with FcϵR1, demonstrating a high affinity (Kd≈10^-10^M) (89). The disparities in binding affinity underscore the functional differences between these antibodies. Hence, as a result of its very slow dissociation, free IgE levels remain very low, with a circulatory half-life of only 2–3 days, in contrast to the roughly 3-week half-life of IgG, and this is mainly because IgE rapidly attaches to cells rather than being degraded (90). Accordingly, several studies have reported that IgE can remain bound to FcϵRI for several weeks (7, 90–92). This long-term binding enables sustained sensitization of mast cells and basophils, a property with important clinical implications. For instance, allergic reactions to peanuts have been observed in organ transplant recipients due to donor-derived, mast cell-bound IgE (93, 94).

These findings highlight the unique structural and functional properties of IgE and underscore the potential for therapeutic interventions targeting the IgE–FcϵRI interaction or its binding kinetics.

### 2.1 Immunoglobulin E glycosylation

Plasma cells, the mature form of B cells, produce immunoglobulins (Igs) as part of the body’s defense against pathogens. Igs and their corresponding Fc receptors are glycoproteins that connect adaptive and innate immune responses. Glycosylation—the attachment of oligosaccharides to the protein backbone—significantly influences the biological functions of antibodies. Two main classes of glycans can attach to proteins: asparagine (N)-linked and serine/threonine (O)-linked glycans (95). N-linked glycosylation is a post-translational modification involving the covalent attachment of a glycan to the asparagine (Asn) residue of a protein within the consensus sequence Asn-X-Ser/Thr/Cys, where X represents any amino acid except proline (96). In the endoplasmic reticulum, 14 glycan units linked to a dolichol phosphate precursor are transferred en bloc to Asn residues in nascent polypeptides (97). These precursor glycans undergo extensive enzymatic modification as they transit through the Golgi apparatus before reaching their intra- and extracellular destinations (98). In mammals, the most common monosaccharides involved in glycan assembly include glucose (Glc), mannose (Man), galactose (Gal), N-acetylglucosamine (GlcNAc), fucose (Fuc), and sialic acid. Most membrane-bound and secreted proteins undergo glycosylation (99).

Unlike the linear structure of protein backbones, which is genetically encoded, glycan structures are shaped by a complex network involving hundreds of glycogenes (100). These genes encode glycosyltransferases, glycosidases, sugar nucleotide biosynthetic enzymes, transporters, transcription factors, and ion channels (101). Consequently, glycan structure and diversity are determined by the expression levels and localization of these enzymes, the availability of glycoprotein substrates, and the supply of activated sugar donors (102). The resulting structural diversity of glycans—due to extensive branching and variable monosaccharide composition—enables them to modulate a wide range of biological processes, including protein folding, cell–cell communication, signal transduction, and immune function (103). Glycosylation is also a key regulator of antibody stability, half-life (104–110) and overall immune activity (111).

Among antibody classes, IgE is the most heavily glycosylated, with oligosaccharides comprising approximately 12% of its total mass (112–114). Glycosylation is essential for IgE secretion (69); however, further processing of its precursor oligosaccharide (Glc_3_Man_9_GlcNAc_2_) is not required for secretion, allergen recognition, or mast cell activation (115). As discussed above, IgE interacts with innate immune cells— MCs, basophils, eosinophils, dendritic cells (DCs), and monocytes,—through binding of its Fc region to the high-affinity FcϵRI receptor on effector cell surfaces (81, 116, 117). However, the role of FcϵRI on some specific DCs remains ambiguous (118). Upon allergen exposure, IgE bound to FcϵRI is crosslinked, leading to effector cell degranulation and the release of proinflammatory mediators such as histamine, prostaglandins, and leukotrienes (6). These mediators promote vasodilation, increased vascular permeability, bronchoconstriction, leukocyte extravasation, and smooth muscle contraction, driving both the acute and late-phase allergic responses (119). In severe cases, this cascade can result in systemic anaphylaxis.

In addition to FcϵRI, IgE also binds the low-affinity receptor FcϵRII (CD23), which is expressed on B cells, macrophages, DCs, eosinophils and platelets (81, 117). FcϵRII (CD23) exists in both membrane-bound and soluble forms (6). Structural studies by German et al. and Holdom et al. revealed that IgE binds to FcϵRI through its Cϵ3 domain, whereas binding to FcϵRII involves both the Cϵ3 and Cϵ4 domains (120, 121). The conformation of IgE determines its receptor interactions: it binds FcϵRI in an open configuration and CD23 in a closed configuration. Binding to one receptor induces conformational changes that prevent simultaneous binding to the other. Moreover, soluble FcϵRIα fragments and soluble CD23 (sCD23) can competitively inhibit IgE binding to both receptors (116, 122, 123).

Recent studies have shown that CD23 is a key IgE receptor that plays an important role in regulating IgE synthesis and mediating immune responses against intracellular pathogens (124–126). Studies have shown that mice lacking CD23 exhibit increased levels of IgE (127). Furthermore, various research studies have shown that IgE, when bound to antigens, has a greater tendency to attach to CD23 on B cells *in vivo*, which in turn promotes the formation of antigen-specific T cells and antibodies (128, 129). Plattner et al. (130) reported that administering antigen-complexed IgE multiple times in mice can induce the formation of protective IgG antibodies that target both antigen-specific and non-specific IgE, with the IgE–IgG complex being cleared in a CD23-dependent manner. Notably, even though Endo F1-treated human IgE and untreated human IgE exhibit similar binding to CD23, mice that received immunization with an Endo F1-treated IgE immune complex exhibited lower levels of anti-IgE–IgG antibodies compared to those immunized with an untreated IgE immune complex (131, 132). These findings suggest a unique CD23-IgE interaction that warrants further examination for the development of new IgE-targeted therapies.

Soluble CD23 increases IgE production, especially after class-switch recombination (133). This circulating receptor acts by binding to cells expressing membrane IgE (mIgE) and membrane CD21 (mCD21), promoting their aggregation on B cells, which is crucial for the successful production of IgE (133). Conversely, increased levels of secreted IgE can bind to mCD23, potentially inhibiting further sCD23 release and thereby maintaining homeostasis (133). While sCD23 enhances IgE synthesis, other studies show that targeting CD23 can inhibit IgE production (126), suggesting a complex regulatory network in which soluble and membrane forms of CD23 may have opposing effects on IgE synthesis. Hence, instead of serving as a low-affinity receptor that regulates IgE synthesis, CD23 may function as a glycan-binding receptor in various mammalian species, including cows and mice (134). The carbohydrate recognition domains (CRDs) of cow and mouse CD23 exhibit specific binding to glycans, including mannose, N-acetylglucosamine (GlcNAc), glucose, and fucose (134). In humans, the absence of glycan-binding activity in CD23 results from evolutionary mutations that disrupt key glycan-binding residues, reducing CD23 function (134). This research shows that CD23 has species-specific glycan-binding properties and highlights its dual role in the immune system as both an IgE regulator and a glycan-binding receptor.

### 2.2 The role of Fc glycans on IgE activity

Human IgE (hIgE) consists of two heavy chains, each containing seven N-glycosylation sites, whereas mouse IgE (mIgE) contains nine. No evidence supports the presence of O-glycosylation sites on IgE. Among these sites, the oligosaccharides attached to Asn394 in humans and Asn384 in mice are oligomannosidic in nature (135). In contrast, five other hIgE glycosylation sites contain complex-type glycans, while one site remains unoccupied (**Figure 1b**).

Extensive studies have characterized IgE glycan structures using IgE purified from monoclonal myeloma cells, recombinant mammalian expression systems, and serum samples from healthy donors or patients with IgE myeloma, atopic dermatitis, or hyper-IgE syndrome. Across all sources, oligomannosidic glycans (Man_9_–Man_9_) have been consistently identified at Asn394 (88, 112, 114, 121, 135–137).

The glycan attached to Asn394 plays a critical role in the synthesis and biological activity of IgE in mammalian cells (138). This site is homologous to Asn297 on the Fc region of IgG1, where N-glycans are also essential for effector functions (139). IgG-Fc glycans are typically biantennary complex-type structures, characterized by core fucosylation and often modified with bisecting N-acetylglucosamine (GlcNAc) (140, 141). These antennae may also carry terminal sialic acid residues with variable galactosylation (142). In IgG, the Fc glycan is embedded within the CH2 domain’s hydrophobic core, forming numerous noncovalent interactions with the polypeptide backbone that stabilize Fc conformation (143, 144). Consequently, IgG glycosylation significantly affects molecular stability and effector function. Physiological and pathological conditions that alter Fc glycan structure can shift IgG effector activity.

Similarly, extensive research has focused on how IgE glycosylation affects its structural and functional properties, particularly its interaction with FcϵRI. Björklund et al. reported that deglycosylation impairs IgE–FcϵRI binding (145), whereas other studies found minimal or no impact (146–148). The Asn394 glycan, located on the Cϵ3 domain of IgE, occupies an interstitial region between the Fc fragments. Enzymatic removal of this glycan using EndoF1 disrupts IgE–FcϵRI binding, analogous to the role of the Asn279 glycan in IgG (135). This conformational alteration prevents FcϵRI engagement and thereby suppresses allergic inflammation.

Mutation studies have confirmed the structural and functional significance of the Asn394 glycan (135, 138, 149), indicating that glycosylation is indispensable for binding to FcϵRI receptors. Genetic disruption of this site abolishes IgE-mediated mast cell degranulation, and analogous modification of the mouse Asn384 residue or total enzymatic deglycosylation by PNGase F eliminates FcϵRI binding *in vivo* and *in vitro* (135). Remarkably, IgE molecules retaining only the Asn384 glycan site can still trigger anaphylaxis comparable to wild-type IgE, indicating that this site alone is essential for IgE effector function (135). In contrast, glycosylation appears less critical for IgE interaction with the low-affinity receptor CD23. Vercelli et al. demonstrated that enzymatic deglycosylation enhances IgE–CD23 binding, suggesting that glycosylation may hinder this interaction (148). Collectively, these findings indicate that IgE N-glycans differentially regulate receptor interactions: loss of glycosylation promotes a closed IgE conformation favoring CD23 binding while simultaneously reducing FcϵRI affinity.

Indeed, to further emphasize the importance of site-specific IgE glycosylation patterns for treatment outcomes, Bohle et al. (132) indicate that the elimination or alteration of the N394 glycosylation site on IgE prevents omalizumab from binding to IgE, thereby hindering its binding to FcϵRI and CD23 and mitigating allergic reactions. When IgE is either deglycosylated or the N394 site is altered, omalizumab is unable to bind or inhibit IgE’s interaction with CD23 (132). This research further potentiates the importance of IgE glycosylation in the design and function of anti-IgE therapies.

Mass spectrometry analyses by Shade et al. (150) revealed that six glycosylation sites are typically occupied in human IgE: Asn140, Asn168, Asn218, Asn265, Asn371, and Asn394. Among these, Asn394 carries oligomannosidic glycans, while the others contain complex-type structures; Asn383 remains unoccupied, consistent with previous studies (114, 136, 137). These complex glycans are primarily fucosylated and sialylated, though their abundance and structure vary across individuals and disease states. Shade et al. reported that non-atopic IgE contains galactose-terminated complex glycans at Asn140 and Asn265, whereas allergic IgE carries disialylated glycans at Asn168 and Asn265 (150).

Similarly, Plomp et al. found elevated mono- and disialylated glycans in non-myeloma samples, while IgE myeloma patients exhibited increased tri- and tetra-antennary structures and reduced bisecting GlcNAc residues (136). These findings underscore the heterogeneity and disease-dependent variation of IgE glycosylation. Such alterations may influence IgE–FcϵR interactions and downstream immune responses. From a therapeutic perspective, engineering glycosylation of recombinant antibodies to enhance or suppress effector function represents an important and active area of research (151). A deeper understanding of IgE Fc glycan roles in pathology could facilitate the development of novel IgE-targeted therapies.

As with other antibody classes, IgE glycans display extensive heterogeneity. For comparison, the conserved Asn297 of IgG Fc can carry up to 36 distinct glycoforms (152). Among these, terminal sialylation has gained significant attention due to its role in modulating antibody activity (153). Increased sialylation converts IgG from a pro-inflammatory to an anti-inflammatory state (152, 154), and this property underlies the therapeutic efficacy of high-dose intravenous immunoglobulin (IVIG) in autoimmune diseases such as immune thrombocytopenia (ITP), chronic inflammatory demyelinating polyneuropathy (CIDP), and rheumatoid arthritis (RA) (152, 155–157). Conversely, less-sialylated IgG forms activate Fcγ receptors, promoting pro-inflammatory responses and contributing to autoimmune disorders such as ITP, autoimmune hemolytic anemia (AHA), systemic lupus erythematosus (SLE), type 1 diabetes, and multiple sclerosis (152, 158, 159).

Shade et al. (150) also highlighted the role of sialylated glycans in IgE-mediated allergy. For instance, desialylation reduced IgE’s ability to induce degranulation without affecting FcϵRI binding or antigen recognition. In parallel, mutating Asn265 and Asn374 did not alter FcϵRI binding or degranulation activity (135, 138). However, when the three N-glycosylation sites within the Cϵ1 domain of the Fab region were mutated, a slight reducetion in degranulation were observed, suggesting that Cϵ1 glycans may influence antigen binding. Expanding on this, enzymatic removal of sialic acid from recombinantly produced human IgE in HEK cells increased binding to CD23-expressing human leukemic B cells *in vitro* (160). Despite these differences in binding, both sialylated and asialylated human IgE antibodies elicited comparable degranulation in a rat basophilic cell line (161). This suggests that sialic acid plays a predominant pathogenic role in IgE-mediated allergic responses in humans. These findings point to a new therapeutic avenue: targeting IgE-binding sialic acids could initiate a transformative phase in the treatment of IgE-dependent allergies. As such, numerous efforts have targeted sialic acid-interacting lectins (SIGLECs) associated with the IgE-FcϵRI receptor complex for therapeutic applications. Notably, nanoparticles coated with ligands specific to Siglec-8 or Siglec-3, along with the corresponding IgE antigen, have been shown to facilitate the recruitment of Siglec-8 or Siglec-3 to the IgE-FcϵRI receptor complex (162, 163), which triggers dephosphorylation of Syk and a reduction in PSA *in vivo*. Moreover, in an *in vitro* setting, the presence of Siglec-3 at the IgE-FcϵRI receptor complex was found to suppress the activation of blood basophils from individuals with peanut allergies when these cells were exposed to peanut extract (164).

Recent studies propose that lectins may modulate IgE function and FcϵRI signaling. Niki et al. demonstrated that galectin-9, a lectin known to inhibit MCs degranulation, binds to IgE glycans and interferes with antigen binding (165). In contrast, galectin-3 can crosslink IgE and FcϵRI, promoting MCs activation (166). Galectin-3 has emerged as a biomarker of allergic disease: Gao et al. reported elevated Gal-3 in eosinophilic asthma, while Riccio et al. identified it as a predictor of favorable responses to omalizumab therapy in severe asthma, correlating with improved airway remodeling and reduced eosinophilic inflammation (167, 168). Recently, Plattner et al. (131) demonstrated that mice immunized with IgE-allergen immune complexes (IgE-ICs) developed glycan-specific anti-IgE autoantibodies. Consequently, these autoantibodies inhibited the sensitization of effector cells, reduced overall IgE levels in the bloodstream, and protected mice from both passive and active IgE sensitization. As a result, this immune response provided cross-protection against various allergens. Furthermore, glycan-specific anti-IgE autoantibodies were found in the sera of both allergic and non-allergic mice (131). Notably, this research demonstrates, in a murine model, that glycan-specific IgG anti-IgE autoantibodies can reduce serum IgE concentration and anaphylactic activity. In a subsequent study, the authors revealed the significant role of glycosylated IgE-ICs in triggering an increased anti-IgE IgG response and a higher production of IgG-secreting plasma cells compared to the deglycosylated IgE-ICs, which showed a marked decrease in IgE clearance and protection of systemic anaphylaxis, indicating that the IgE glycans themselves are the main contributors to the protective effect induced by the IgE-ICs and could further mediate a strong anti-IgE IgG response and control of serum IgE levels (130).

Despite growing evidence, the biological roles of complex glycans in IgE remain less defined than in IgG. Further investigation is needed to elucidate how site-specific glycosylation regulates IgE structure, receptor binding, and immune function, paving the way for glycoengineering approaches to treat allergic and immune-mediated diseases.

## 3 IgE immune protection role in parasitic infections

Parasitic infections remain a major public health concern in tropical and subtropical regions. According to the World Health Organization, approximately one billion people are infected with various helminth species across sub-Saharan Africa, Asia, and the Americas, imposing a significant burden on healthcare systems and socioeconomic development (169). Bethony et al. estimated that over 25% of the global population is infected with helminths such as *Ascaris lumbricoides (roundworm)*, *Trichuris trichiura (whipworm), Necator americanus* and *Ancylostoma duodenale (hookworms), schistosomes*, and *filarial worms* (170). Although mortality from these infections is relatively low, helminthiasis contributes substantially to morbidity through anemia, malnutrition, and impaired growth and cognition (171). Because helminths are large and often migrate through host tissues, the nature of host defense varies depending on the specific parasite species (172).

Poor sanitation, limited access to clean water, and inadequate hygiene practices are primary drivers of chronic helminth infections in rural regions (173, 174). Interestingly, allergic diseases and asthma have also become increasingly prevalent in both developing and industrialized countries, particularly in urban populations (175–179). These epidemiological observations suggest that altered environmental exposures, in combination with genetic predisposition, contribute to the rise of allergic disorders. The higher incidence of allergic diseases in urban environments has been attributed to reduced exposure to childhood pathogens, smaller family sizes, and lifestyle changes (180).

A growing body of evidence links the global increase in allergic diseases to diminished exposure to helminths during early life (181). Chronic helminth infections can modulate host immunity, promote an anti-inflammatory environment and generalized T-cell hyporesponsiveness (182). This phenomenon is encapsulated by the “hygiene hypothesis,” which proposes that reduced exposure to microbes and parasites in childhood increases susceptibility to allergic and autoimmune diseases (182–184). Experimental studies have shown that helminth administration can suppress autoimmune and allergic inflammation, whereas deworming may exacerbate these conditions (182, 185, 186). Understanding the regulatory mechanisms—particularly anti-inflammatory pathways—induced by helminth infection could provide valuable insights for developing novel therapies against immune-mediated diseases.

Helminth infections and allergic diseases share several immunological features, most notably the induction of Th2-type immune responses characterized by elevated Th2 cytokines, IgE production, and activation of effector cells (187, 188). These responses are associated with increased secretion of IL-4, IL-5, IL-9, IL-13, and IL-21 (189). IL-25 also plays a critical role in Th2 immunity and helminth expulsion by promoting IL-5 and IL-13 expression (190, 191). Although IL-10 is often categorized as a Th2-type cytokine (192, 193), it acts broadly as an immunosuppressive mediator that inhibits both Th1 and Th2 responses via regulatory T cells (194). Thus, cytokines central to Th2 responses (e.g., IL-4, IL-13, IL-21, and IL-25) can simultaneously downregulate Th1- and Th17-type inflammation (189).

**Figure 2:**
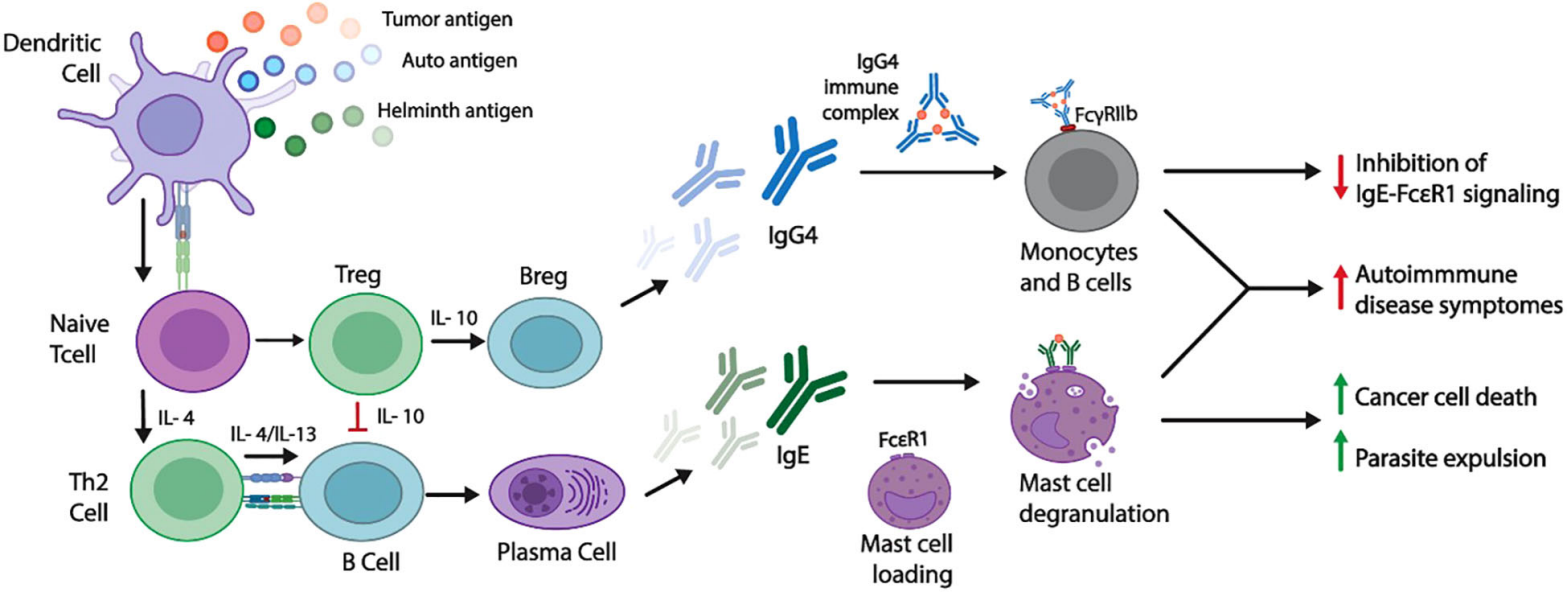
Schematic presentation of IgE-mediated immune response mechanism in non-atopic disorders.Non-atopic disorders activate specific innate immune system cells known as activated APCs, including DCs. Since the role of DCs is to bind antigens, they transport those antigens to lymph nodes and present them to naïve T cells. Consequently, DC induces Th2 responses, which initiate the release of specific cytokines (IL-4 and IL-13). These cytokines activate signaling pathways on B cells and help the production of antigen-specific IgE. Cross-linking antigen-specific IgE antibodies with the FcϵRI receptors on effector cells leads to degranulating these innate immune cells. Respectively, several proinflammatory mediators like histamine, tryptase, prostaglandins, and leukotrienes will be released, initiating protection against helminth infections, preventing tumor growth, and increasing antitumor immunity (196). Chronic helminth infection promotes responses such as IL-10, regulatory T cells, and regulatory B cells, which can prevent Th2 responses’ downstream effector phase. Increased levels of suppressive IL-10 cytokines can reduce the production of IgE and promote a switch to IgG4, a type of immunoglobulin not associated with clinical allergies. It has been suggested that IgG allergen complexes could inhibit signaling through the IgE FcϵRI pathway by binding to the Fc receptor (FcγRIIb) (197). MCs and basophils are known to play roles in antibody-mediated diseases (AAID) where autoreactive IgE and FcϵRI aggregating antibodies are present. These antibodies have been implicated in the onset and progression of diseases.

IgE also plays a pivotal role in host defense. Parasite-specific IgE binds to high-affinity FcϵRI receptors on MCs and basophils or to low-affinity CD23 receptors on eosinophils, macrophages, DCs, and B cells, promoting parasite recognition and clearance (6, 195). Upon FcϵRI engagement, IgE triggers effector cell degranulation and the release of bioactive mediators that facilitate parasite expulsion (**Figure 2**) (189, 198). Despite the shared immune mechanisms between helminth infection and allergy, their clinical outcomes differ: IgE-mediated allergic reactions are typically pathological, whereas IgE responses to helminths are protective (199, 200). Numerous studies have demonstrated positive correlations between parasite-specific IgE levels and resistance to infection, supporting a protective role for IgE in helminth immunity (201–207). The first *in vivo* evidence came from studies showing that passive transfer of monoclonal IgE specific to *Schistosoma* antigens conferred protection (208). Similarly, elevated parasite-specific IgE levels in *Schistosoma haematobium*–infected individuals correlated with reduced reinfection rates (205), and IgE responses to *S. mansoni* were associated with enhanced resistance (209).

Nkurunungi et al. (210) showed that rural participants from *S. mansoni*-endemic islands had higher IgE and IgG responses to parasite glycan antigens, including the core β-1,2-xylose and α-1,3-fucose N-glycans, whereas urban participants with less exposure had weaker responses. In addition, in rural areas, individuals infected with *S. mansoni* had increased glycan-specific IgE responses to active S. mansoni compared to uninfected controls. This research may indicate that IgE undergoes glycosylation-driven structural changes to detect distinct parasite glycans. To further investigate the role of antibody glycosylation in parasite glycan recognition, Adjobimey and Hoerauf showed that chronic helminth infections increase sialylation and bisecting GlcNAc on IgG, possibly indicating a similar mechanism in the IgE antibody response (211). These findings underscore the need for further research into how antibody glycosylation variability influences immune responses and allergy diagnostics in helminth-endemic areas.

It is well known that parasites manifest cross-reactive carbohydrate determinants (CCDs), such as core α1,3-fucose and β1,2-xylose, that mimic host IgE glycans. Additionally, specific glycans attached to helminths promote an IgG-associated Th2 Immune response that mitigates IgE-mediated immunity (160). In this sense, parasites may utilize a mechanism to avoid immune surveillance by exploiting glycosylated IgE-ICs that stimulate the generation of glycan-specific IgG autoantibodies, thereby facilitating the removal of IgE from circulation (130). Furthermore, several studies using monoclonal IgE have indicated that, following parasitic infection, certain parasite antigens, such as excretory/secretory proteins, serve as key immunogenic targets to hinder parasite infection and survival (212, 213), implying a central role of IgE in precisely targeting and neutralizing parasitic threats.

Conspicuously, the immune mechanisms elicited by different helminths vary considerably. Both *Heligmosomoides polygyrus (a nematode)* and *Schistosoma mansoni (a trematode)* induce Th2-type immunity in mice, yet their protective strategies differ. In *H. polygyrus* infection, the Th2 response causes stress and expulsion of the parasite (214), while in *S. mansoni* infection, Th2 cytokines mitigate Th1-mediated immunopathology rather than eliminate the parasite (215). These examples illustrate that Th2-driven responses may serve either parasite clearance or immune regulation, depending on the infection context (189). Such heterogeneity in parasite biology, infection intensity, timing, and host genetics likely contributes to the variable epidemiological associations between helminth infections and allergic diseases (216).

For example, *Ascaris lumbricoides* infection has been linked to increased asthma risk, whereas hookworm infection shows a protective association (217). Other intestinal parasites, such as *Trichuris trichiura*, *Enterobius vermicularis*, and *Strongyloides stercoralis*, appear to have no significant effect (217). Since the 1970s, many studies have explored the modulatory effects of helminth infections on allergy and asthma (218–222), with most suggesting that helminths reduce allergic sensitization (223). One proposed mechanism is that helminths induce strong polyclonal IgE production, which elevates total serum IgE and may competitively inhibit allergen-specific IgE binding and effector cell activation (224). This polyclonal activation may represent an immune evasion strategy that allows parasites to avoid host detection (224).

Children with atopic backgrounds have been shown to mount stronger immune responses to helminths yet exhibit lower infection intensities than non-atopic children, suggesting a potential evolutionary advantage of atopy in resisting infection (224). However, Mitre et al. found that the ratio of polyclonal to allergen-specific IgE did not suppress basophil degranulation (225). Elevated IgE levels can upregulate FcϵRI expression, while anti-IgE therapy reduces receptor density, indicating that high total IgE may not effectively compete with allergen-specific IgE for receptor binding (226).

Yazdanbakhsh et al. proposed that some parasite infections induce allergen-specific IgE of low biological activity, incapable of triggering effector cell activation (180). Supporting this, several studies reported that increased IgE targeting CCDs exhibits low functional activity (227, 228). Another mechanism involves helminth-induced production of IgG4, which can compete with IgE for allergen binding. IgG–allergen complexes may engage the inhibitory FcγRIIb receptor, activating phosphatases that dampen IgE–FcϵRI signaling (**Figure 2**) (197, 229–231).

Recent evidence also highlights IL-10 as a central regulator during chronic helminth infection. Elevated IL-10 levels attenuate basophil responsiveness to IgE stimulation (232), suppress T- and B-cell activation (233), enhance IgG synthesis (234), and promote B-cell differentiation toward IgG4 production (235–237). Collectively, these mechanisms demonstrate that chronic helminth infections induce potent immunoregulatory activity mediated by IL-10 and regulatory T cells (Tregs), which suppress Th2 responses and reduce inflammatory pathology (**Figure 2**) (238, 239). Hence, it is well established that parasitic diseases elicit strong IgE responses in infected individuals, but IgE-based diagnostics remain ambiguous. New sensitive IgE tests and synthetic peptides now enable better detection of parasite-specific IgE, potentially improving the accuracy of these blood tests (240, 241). Building on these diagnostic developments, it is also important to consider related therapeutic interventions. Therapeutically, monoclonal antibodies targeting IgE, like omalizumab, are used in allergic diseases; their impact on susceptibility to parasitic infections warrants further study (242, 243).

## 4 IgE in autoimmunity

Studies estimate that autoimmune diseases affect 7–9% of the global population (244, 245). Recent research into gender-based immunological differences reveals that women have a higher incidence of autoimmune diseases than men (246, 247). Central to preventing autoimmunity is immune tolerance, which protects the body from attacking its own antigens (248). When tolerance is breached, the immune system mounts a strong response against self-antigens, leading to autoimmune pathology (248). Autoimmune diseases arise from failures in multiple self-reactivity control mechanisms, involving the activation of innate immune molecules that recognize self or foreign antigens (245).

Regulatory T cells (Tregs), regulatory B cells, their suppressive cytokines, and surface molecules are crucial for maintaining self-tolerance. Dysregulation or loss of function in these cells, due to physiological changes, can promote allergic and autoimmune diseases (249, 250). Additionally, reduced Treg number and function—potentially resulting from decreased exposure to chronic infections—increases Th1 and Th2 activity, raising the risk of these disorders (251, 252).

While allergen-specific IgE antibodies contribute to allergic disease pathogenesis by promoting Th2 immunity, self-reactive IgE also plays a role in autoimmune tissue damage, a hallmark of autoimmunity (253). Interestingly, elevated total or autoreactive IgE levels do not always correlate with increased allergic disease incidence, highlighting the complex role of IgE dysregulation in inflammation (254–256).

B cells contribute to autoimmunity by producing IgE, presenting antigens, and releasing cytokines (250). Plasma B cell proliferation and differentiation depend on IL-6, a potent B-cell activating factor (253). B cells can generate all autoantibody subclasses, including IgE, which initiate autoimmune reactions by binding FcϵR receptors on effector cells. Although the role of IgE in non-atopic disorders is not fully understood (257), evidence indicates that interactions between IgE autoantibodies and FcϵRI receptors on mast cells (MCs) and basophils are key in triggering autoimmune symptoms (**Figure 2**) (258). Basophils, when activated by IgE-autoantigen complexes, can further promote the differentiation of B cells and the production of autoantibodies, creating a self-perpetuating cycle (259).

Beyond its established role in allergic diseases, IgE’s involvement in autoimmune conditions has gained attention (258). Pathogenic IgE has been implicated in rheumatoid arthritis (RA) (260, 261), bullous pemphigoid (BP) (262), atopic dermatitis (AD) (263), systemic lupus erythematosus (SLE) (264), uveitis (265), systemic sclerosis (266), multiple sclerosis (267), Hashimoto thyroiditis, and Graves disease (268, 269), and chronic spontaneous and inducible urticaria (270, 271). These diseases show immune responses mediated by specific IgE autoantibodies, supporting IgE’s role in autoimmunity (253).

SLE, a systemic autoimmune disorder affecting multiple organs (272, 273), is characterized by immune dysfunction and typical lab findings including hypergammaglobulinemia and IgG antinuclear antibodies (253). Elevated serum IgE levels correlate positively with disease activity in SLE patients (255, 274–276). Henault et al. demonstrated that IgE autoantibodies specific for double-stranded DNA (dsDNA) activate plasmacytoid dendritic cells (pDCs), triggering high interferon-α (IFN-α) release, which amplifies autoimmune damage (19, 277). Anti-dsDNA IgE also enhances pDC phagocytosis via FcϵRI binding, activating Toll-like receptor 9 (TLR9) signaling (277).

IFN-α has been shown to suppress eosinophil granule protein secretion (278) and mast cell histamine release (279). More recently, IgE cross-linking on pDCs inhibits Treg synthesis *in vitro*, an effect reversed by omalizumab, an anti-IgE monoclonal antibody (245, 258). Omalizumab restores pDC function and Treg homeostasis, suggesting its potential as a treatment for autoimmune diseases with impaired Treg activity.

Unlike allergic diseases, where IgE mediates hypersensitivity, IgE in autoimmune diseases like SLE appears to engage interferon-driven responses to nucleic acids (258). Studies show no increased prevalence of allergic diseases among SLE patients despite elevated IgE (280, 281). Both IgG and IgE autoantibodies share biological activity in SLE, with only specific IgE autoantibodies binding nucleic acids directly or indirectly (282).

Approximately half of SLE patients develop renal complications (283). Anti-dsDNA IgG autoantibodies are well-established diagnostic markers for SLE (253). Dema et al. found a strong association between elevated anti-dsDNA IgE and disease severity, including lupus nephritis (284). Henault et al. confirmed anti-dsDNA IgE as an independent risk factor for SLE activity, regardless of IgG levels (277). IgE autoantibodies also recognize novel autoantigens (APEX nuclease 1, N-methylpurine DNA glycosylase, CAP-Gly domain-containing protein family member 4) not targeted by IgG (284). Pan et al. linked elevated peripheral basophil activity with increased IgE autoantibody production and SLE severity; co-culture experiments showed basophils enhance autoreactive IgE production and promote Th17 differentiation from naïve CD4+ T cells (285). Preclinical mouse models show that IgE deficiency can attenuate lupus-like disease, supporting causality. Notwithstanding the lack of conclusive clinical investigation of anti-IgE strategies in SLE, research on modulating pDCs to reduce IFN signatures increased by IgE complexes is especially relevant for SLE (259, 286, 287).

IgE autoantibodies also contribute to chronic spontaneous urticaria (CSU), an autoimmune mast cell-driven disease characterized by hives and angioedema lasting more than six weeks (288). CSU patients exhibit IgG and IgE autoantibodies against FcϵRI, dsDNA, thyroglobulin, and thyroperoxidase (289–292).

Elevated anti-dsDNA IgE in CSU does not correlate with anti-dsDNA IgG levels (293). Notably, IgE autoantibodies targeting the cytokine IL-4 and IL-24 have been linked to CSU severity (294). Omalizumab is a well-established treatment for CSU, and its response patterns can help in differentiating between autoimmune and autoallergic pathways (295, 296). Maurer et al. demonstrated that omalizumab effectively reduces symptoms in CSU patients with anti-thyroid peroxidase (TPO) IgE autoantibodies (297). Furthermore, omalizumab-treated patients with IgE-mediated CSU show faster symptom relief compared to those with IgG-mediated CSU (298).

Bullous pemphigoid (BP) is an autoimmune blistering disease targeting hemidesmosomal proteins BP230 and BP180 in the skin’s dermal-epidermal junction (299, 300). BP serves as a key model for studying IgE-mediated autoimmunity (301). Recent cohort and mechanistic studies continue to highlight the role of IgE in the pathogenesis of BP, showing that a considerable number of patients exhibit measurable levels of anti-BP IgE (302–305). IgE autoantibodies bind BP180, cross-link FcϵRI on mast cells and basophils, triggering degranulation and inflammation (306–308). Approximately 70–90% of BP patients have both IgG and IgE autoantibodies targeting BP180, with levels correlating with disease severity (308, 309). IgE binding to BP180 on keratinocytes induces antigen internalization, release of IL-6 and IL-8, and basement membrane disruption (301).

A recent study indicates that IgE autoantibodies can directly activate keratinocytes and other cells located within tissues, contributing to the onset of organ-specific autoimmune disorders (310). Bao and colleagues, in their study of a preclinical mouse model of BP, demonstrated that deleting Myd88 in Krt14 cells greatly reduces disease severity and lowers serum IL-4 and IL-9. This study shows that keratinocyte-driven inflammation drives the systemic response in BP, highlighting that keratinocytes mediate the effects of autoantibodies (310). It is also reported that IgE-ICs can accumulate in tissues such as the skin and the renal glomeruli, thereby indirectly activating complement or initiating inflammatory pathways that bypass FcϵRI (311). Hence, IgE-ICs can directly affect keratinocytes in BP, extending the mechanistic scope beyond the typical type I allergic responses (311). Omalizumab treatment reduces IgE levels and improves symptoms in BP patients (301). Some studies also report IgE autoantibodies against BP230 without BP180 involvement (312–314).

In summary, beyond its well-known role in allergic inflammation, IgE has emerging and diverse functions in autoimmune diseases. Although IgE plays a pathogenic role in autoimmunity, it also regulates the immune response. For example, natural anti-IgE autoantibodies—present even in healthy individuals—may help regulate IgE activity and maintain immune homeostasis (243, 315). Furthermore, IgE can influence adaptive immune responses by modulating antigen presentation and Treg function. As a result, dysregulation of these processes may contribute to the breakdown of self-tolerance and the development of autoimmunity (21, 258, 315). Besides, activation of FcϵRI by IgE can drive inflammation without causing classic allergic reactions, underscoring the complexity of IgE biology and highlighting the need for continued research to unravel its multifaceted roles in immune regulation. Collectively, these data indicate that it is imperative to stratify patients to identify those most likely to benefit from IgE-targeted therapy (296). Future improvements in assay standardization, mechanistic investigations, and controlled therapeutic trials—especially those focusing on anti-IgE treatments—will determine the role of IgE-targeted approaches in standard protocols for autoimmune diseases (19, 311).

## 5 The immunosurveillance role of IgE in cancer

Immunosurveillance, the immune system’s ability to detect and eliminate cancer cells, is essential for preventing tumor development. The host’s immune repertoire can mount robust responses against tumor-specific antigens, potentially influencing clinical outcomes. However, tumors often evade immune detection by manipulating the tumor microenvironment, which suppresses effective immune responses (316). A hallmark of cancer is its ability to escape immune recognition and control. For example, Karagiannis et al. reported that elevated IgG4 levels in melanoma patients correlate with poorer survival, suggesting that tumors may promote IgG4 synthesis as a novel immune escape mechanism (317). Similarly, Andreu et al. showed that neoplastic cells can evade humoral immunity by upregulating inhibitory Fc γ receptors and recruiting specific leukocyte subsets that neutralize therapeutic IgG antibodies (318).

Recently, IgE antibodies have gained attention for their potential role in tumor immunosurveillance and as therapeutic agents (15). Immunohistochemical analysis of advanced head and neck squamous cell carcinomas showed a higher prevalence of IgE-positive cells compared to normal mucosa (319). Most IgE-positive cells had morphological features of plasma cells, suggesting a potential role for IgE in antitumor immunity (319). Fu et al. demonstrated that high IgE levels promote antibody-dependent cellular cytotoxicity (ADCC) against pancreatic cancer cells (320). Multiple studies support that antigen-specific IgE binds to FcϵRI receptors on effector cells to mediate antitumor responses (**Figure 2**) (321–324). IgE binding to its receptors induces tumor-associated macrophages (TAMs) and monocytes to secrete high levels of cytokines, such as TNF-α, IL-1β, and MCP-1, which enhance ADCC against tumor cells (325–328). Genomic and transcriptomic analyses indicate that components of the IgE receptor pathway, including FCER1G, are upregulated in various tumor types and are associated with immune cell infiltration, prognosis, and response to immunotherapy (329). As a result, IgE-mediated immune responses may also limit immunosuppressive interactions between macrophages and regulatory T cells, thereby promoting anti-tumor immunity (327). In addition, IgE also induces antigen cross-presentation by DCs, primes cytotoxic T lymphocyte responses, and supports the development of long-term tumor immunosurveillance (330). Although the protective role of allergic diseases and IgE in cancer has been documented, the exact mechanisms by which allergy-driven inflammation influences tumor development remain unclear and warrant further study (331). The emerging field of allergooncology focuses on IgE-mediated tumor destruction and the development of IgE-based immunotherapies, offering new perspectives and potential treatments for cancer (332, 333).

Epidemiologic studies indicate that individuals with allergic symptoms and elevated IgE levels have a reduced risk of various cancers, including childhood leukemia, pancreatic, brain, ovarian, colorectal, glioma, and gynecological cancers (319, 331, 332, 334–336). Conversely, ultra-low IgE levels may be a biomarker for increased cancer risk (337, 338). Ferastraoaru et al. reported a significant association between selective IgE deficiency and increased cancer risk in both adults and children (339–341). Specific IgE antibodies against tumor-associated antigens have been detected in human serum (320, 342), and mouse models confirm IgE’s protective role against several tumors (321, 343, 344). Although the precise immunological mechanisms remain unclear, current evidence suggests that IgE can mediate tumor cell eradication and hold promise as an anticancer agent (345).

Effector cells expressing FcϵR receptors within the tumor microenvironment face an immunosuppressive milieu that promotes tumor growth, invasion, and progression (346). Yet, immune cell infiltration into tumors correlates with reduced recurrence and improved survival, reflecting ongoing immune surveillance (347). Targeting tumors via IgE-FcϵR interactions on effector cells presents a promising therapeutic strategy. Antigen-specific IgE antibodies against targets like folate receptor alpha (FRα) and HER2/neu have shown enhanced anticancer efficacy by engaging FcϵR-expressing effector cells in the tumor microenvironment (322–324, 348, 349). Notably, MOv18 IgE antibodies targeting FRα improved survival and limited ovarian carcinoma progression more effectively than IgG1 in animal models (322, 324, 348).

MCs accumulate in tumors and their microenvironment, influencing innate and adaptive immunity (350, 351). While MCs promote angiogenesis and are linked with poor prognosis in cancers such as melanoma, pancreatic adenocarcinoma, and colorectal cancer (352–360), they can also enhance antitumor responses in mesothelioma, breast cancer, and other malignancies (361, 362). MC degranulation can impair regulatory T cell activity and promote cytotoxic T lymphocyte (CTL)–mediated tumor cell killing through tumor necrosis factor α (TNF-α) release (363, 364). MCs incubated with anti-CD20 IgE antibodies induce lymphoma cell death, demonstrating IgE’s capacity to stimulate effector cells to eradicate tumor cells (365).

Eosinophils, abundant in tumor tissues (tumor-associated tissue eosinophilia, TATE), correlate with better prognoses in various cancers, including esophageal, colorectal, gastric, and Hodgkin lymphoma (366–371). They promote tumor immunity through antibody-dependent mechanisms and modulation of the tumor microenvironment, although their exact protective pathways are not fully understood (370, 372–374). Eosinophil degranulation releases cytotoxic proteins like major basic protein (MBP), contributing to tumor cell death (375). Tumor antigen-specific IgE enhances eosinophil-mediated cytotoxicity, particularly in allergic individuals (324, 376). Basophils, like MCs, express FcϵRI and secrete Th2 cytokines, histamine, and other mediators upon activation, playing key roles in IgE-mediated immune responses and immunotherapies (188).

Recent studies highlight a novel mechanism by which DCs utilize IgE-FcϵRI interactions for cross-presentation of tumor antigens, activating cytotoxic CD8+ T lymphocytes (CTLs) even at low antigen doses. This pathway is independent of MyD88 and IL-12 signaling (330). Passive immunization with tumor-specific IgE and DC-based vaccines enhances antitumor immunity and generates durable memory responses *in vivo* (330). Interestingly, IL-4—a Th2 cytokine—can inhibit this IgE-mediated cross-presentation, suggesting a feedback mechanism that modulates CTL responses during allergic reactions (377). This IgE-FcϵRI-mediated activation of DCs within the tumor microenvironment may promote robust and lasting adaptive immunity against tumors.

## 6 Conclusion

Atopic diseases are well-recognized as classical examples of IgE-mediated pathology. However, the role of IgE in non-atopic disorders has recently garnered significant attention within the scientific community. IgE antibodies are increasingly implicated in diseases marked by immune dysregulation, prompting a broader investigation into their diverse immunological functions. While antibody-mediated inflammatory responses play a crucial role in defending against infections and malignancies, dysregulation of these mechanisms can contribute to autoimmune and other pathological conditions.

Glycosylation profoundly influences the structure and function of IgG antibodies, critically modulating their effector activities. Physiological and pathological conditions can alter IgG Fc glycan composition, significantly impacting antibody function (378). This variability is linked to disease outcomes in conditions such as latent Mycobacterium tuberculosis infection (379), rheumatoid arthritis (105), responses to influenza vaccination (380), dengue hemorrhagic fever (381), and granulomatosis with polyangiitis (382). Notably, IgG exhibits greater glycan heterogeneity than the overall plasma glycome (383), endowing it with unique functional capabilities.

Similarly, IgE antibodies initiate potent effector functions through binding to FcϵRI receptors on effector cells. The Fc region of IgE immobilizes the antibody on these cells, enabling prolonged antigen recognition. Like IgG, IgE Fc N-glycan composition varies between individuals and influences biological activity (384). Despite IgE’s central role in allergic reactions, the correlation between total or allergen-specific IgE levels and clinical allergy is inconsistent (385, 386), with antigen-specific IgE also detected in asymptomatic individuals (385, 387). Recent studies have identified specific glycans at Asn394 and Asn384 in the IgE C3 domain that enhances its ability to trigger allergic responses (135). These oligomannose glycans represent promising targets to modulate allergic reactions therapeutically (135).

Changes in IgE site-specific glycosylation may also underline its pathogenic roles beyond allergies and atopic diseases. Given IgE’s involvement in non-atopic conditions, understanding variations in its glycosylation patterns is critical to defining molecular disease signatures and identifying new therapeutic targets. Further glycoproteomic analyses of IgE glycosylation across diverse pathophysiological states could clarify how these modifications influence disease progression, pinpoint specific Fc glycans associated with pathology, and elucidate their biological functions. This knowledge may enable the design of antibodies with tailored glycan structures and enhanced therapeutic efficacy in both *in vivo* and *in vitro* settings.

## Glossary

AAID: Antibody-mediated autoimmune diseases
AD: Atopic Dermatitis
ADCC: Antibody Dependent Cellular Cytotoxicity
AE: Angioedema
AHA: Autoimmune Hemolytic Anemia (AHA)
Anti-dsDNA: Anti-Double-Stranded DNA
APCs: Antigen-Presenting Cells
ASN: Asparagine
BP: Bullous Pemphigoid
BP180: Bullous Pemphigoid 180
BP230: Bullous Pemphigoid 230
Bregs: Regulatory B cells
CH: Constant Heavy Chain
CIDP: Chronic Inflammatory Demyelinating Polyneuropathy
CindU: Chronic Inducible Urticaria
CSR: Class Switch Recombination
CSU: Chronic Spontaneous Urticaria
CTL: Cytotoxic T lymphocyte
Cγ2: Constant Gama Domain 2
Cϵ2: Constant Epsilon Domain 2
DC: Dendritic Cell
dsDNA: Double-stranded DNA
EndoF1: Endoglycosidase F1
Fab: Antigen Binding Fragment
FAP: Facilitated Antigen Presentation
Fc: Crystallizable Fragment
FcγRs: Crystallizable Fragment Gama Receptors
FcγRIIb: Crystallizable Fragment Gama Receptors IIb
FcϵRI: Crystallizable Fragment Epsilon Receptors I
FcϵRII: Crystallizable Fragment Epsilon Receptors II
GlcNAc: N-Acetylglucosamine
HER2/neu: Human Epidermal Growth Factor Receptor 2
hIgE: Human Immunoglobulin E
HL: Hodgkin Lymphoma
HR: Hazard Ratio
IFN-α: Interferon-α
Ig: Immunoglobulin
IgA: Immunoglobulin A
IgD: Immunoglobulin D
IgE: Immunoglobulin E
IgG: Immunoglobulin G
IgG1: Immunoglobulin G1
IgG4: Immunoglobulin G4
IgM: Immunoglobulin M
Igs: Immunoglobulins
IL-2: Interleukin 2
IL-4: Interleukin 4
IL-5: Interleukin 5
IL-6: Interleukin 6
IL-8: Interleukin 8
IL-9: Interleukin 9
IL-10: Interleukin 10
IL-13: Interleukin 13
IL-17: Interleukin 17
IL-21: Interleukin 21
IL-24: Interleukin 24
IL-25: Interleukin 25
ITP: Immune Thrombocytopenia
IVIG: Intravenous Immunoglobulins
JAK3: Janus Kinase 3
mAb: Monoclonal Antibody
MCHII: Major Histocompatibility Complex Class I
MCs: Mastocytes
mIgE: Mouse Immunoglobulin E
mbIgE: Membrane Bound Immunoglobulin E
NMR: Nuclear Magnetic Resonance
pDCs: Plasmacytoid Dendritic Cells
RA: Rheumatoid Arthritis
sCD23: Soluble CD23
sIgE: Soluble Immunoglobulin E
SLE: Systemic Lupus Erythematosus
STAT6: Signal Transducer and Activator of Transcription 6
TATE: Tumor-Associated Tissue Toll-Like Receptor 9
TNF-α: Tumor Necrosis Factor Alpha
Tregs: Regulatory T cells
VL: Variable Light Chains
WT: Wild-Type.
